# Preparation and characterization of spindle-like Fe_3_O_4 _mesoporous nanoparticles

**DOI:** 10.1186/1556-276X-6-89

**Published:** 2011-01-17

**Authors:** Shaofeng Zhang, Wei Wu, Xiangheng Xiao, Juan Zhou, Feng Ren, Changzhong Jiang

**Affiliations:** 1 Key Laboratory of Artificial Micro- and Nano-structures of Ministry of Education, Wuhan University, Wuhan 430072, P. R. China; 2 Center for Electron Microscopy and School of Physics and Technology, Wuhan University, Wuhan 430072, P. R. China

## Abstract

Magnetic spindle-like Fe_3_O_4 _mesoporous nanoparticles with a length of 200 nm and diameter of 60 nm were successfully synthesized by reducing the spindle-like α-Fe_2_O_3 _NPs which were prepared by forced hydrolysis method. The obtained samples were characterized by transmission electron microscopy, powder X-ray diffraction, attenuated total reflection fourier transform infrared spectroscopy, field emission scanning electron microscopy, vibrating sample magnetometer, and nitrogen adsorption-desorption analysis techniques. The results show that α-Fe_2_O_3 _phase transformed into Fe_3_O_4 _phase after annealing in hydrogen atmosphere at 350°C. The as-prepared spindle-like Fe_3_O_4 _mesoporous NPs possess high Brunauer-Emmett-Teller (BET) surface area up to ca. 7.9 m^2 ^g^-1^. In addition, the Fe_3_O_4 _NPs present higher saturation magnetization (85.2 emu g^-1^) and excellent magnetic response behaviors, which have great potential applications in magnetic separation technology.

## Introduction

In the past few decades, porous materials have been used in many fields, such as filters, catalysts, cells, supports, optical materials, and so on [1-3]. In general, porous materials can be classified into three types depending on their pore diameters, namely, microporous (<2 nm), meso- or transitional porous (2-50 nm), and macroporous (>50 nm) materials, respectively [4]. Currently, the mesoporous materials have attracted growing research interests and have great impact in the applications of catalysis, separation, adsorption and sensing due to their special structural features such as special surface area and interior void [2,5-8]. On the other hand, iron oxide nanomaterials have been extensively studied by material researchers in recent years, due to their novel physicochemical properties and advantages (high saturation magnetization, easy synthesis, low cost, etc.) and wide applications in many fields (magnetic recording, pigment, magnetic separation, and magnetic resonance imaging, MRI) [9-16].

However, it is crucial to realize the magnetic iron oxide materials with mesoporous structure which can further adjust the physical and chemical properties of iron oxides for expanding application. According to the previous studies, the porous iron oxide nanomaterials have remarkable magnetic properties, special structures and greatly potential applications in targetable or recyclable carriers, catalyst and biotechnology [17,18]. For example, Yu et al. [19] fabricated novel cage-like Fe_2_O_3 _hollow spheres on a large scale by hydrothermal method. In the report carbonaceous polysaccharide spheres were used as templates, and the prepared Fe_2_O_3 _hollow spheres exhibit excellent photocatalytic activity for the degradation of rhodamine B aqueous solution under visible-light illumination. Wu et al. [20] successfully developed porous iron oxide-based nanorods used as nanocapsules for drug delivery, and this porous magnetic nanomaterial exhibited excellent biocompatibility and controllability for drug release.

It is well known that the intrinsic properties of an iron oxide nanomaterial are mainly determined by its size, shape, and structure. A key problem of synthetically controlling the shape and structure of iron oxide nanomaterials has been intensively concerned by many researchers. In previous studies, there have been various porous iron oxide nanomaterials, such as porous α-Fe_2_O_3 _nanorods, Fe_3_O_4 _nanocages, and so on [9,21-25]. However, to our best knowledge, there are few reports for fabricating the mesoporous structure of monodisperse spindle-like Fe_3_O_4 _NPs. Thus, we employ forced hydrolysis method to prepare spindle-like α-Fe_2_O_3 _NPs first. Then as-prepared α-Fe_2_O_3 _NPs were reduced by hydrogen gas at different temperatures. The structure, morphology, and magnetic properties of samples were investigated by multiple analytical technologies. The results reveal that spindle-like Fe_3_O_4 _mesoporous NPs could be obtained after annealing at 350°C.

## Experimental section

### Materials

Ferric chloride hexahydrate (FeCl_3_·6H_2_O) was purchased from Tianjin Kermel Chemical Reagent CO., Ltd. (Tianjin, China), ethanol (C_2_H_5_OH, 95% (v/v)) and sodium dihydrogen phosphate dihydrate (NaH_2_PO_4_) were purchased from Sinopharm Chemical Reagent Co., Ltd. (Shanghai, China), and all regents used were analytically pure (AR) and as received without further purification. The used water was double distilled water.

### Synthesis of α-Fe_2_O_3 _and Fe_3_O_4 _NPs

Forced hydrolysis method is normally used for the synthesis of α-Fe_2_O_3 _NPs [26]. In the typical procedure, NaH_2_PO_4_·2H_2_O (0.0070 g) was dissolved into 100 ml of water. After completely dissolving, the solution was transferred to a flask (100 ml) and heated to 95°C. Then 1.8 ml of FeCl_3 _solution (1.48 mol l^-1^) was added dropwise into the flask, and the mixture was aged at 100°C for 14 h. After the resulting mixture was cooled down to room temperature naturally, the product was centrifuged and washed with double distilled water and ethanol. The as-obtained α-Fe_2_O_3 _NPs was labeled as S1. The dried α-Fe_2_O_3 _powder was annealed at 250, 300, 350, 400, and 450°C in hydrogen atmosphere for 5 h. These annealed powders were labeled as S2, S3, S4, S5, S6, respectively. All the samples were dispersed into ethanol solution.

### Characterization

XRD patterns of the samples were obtained by using an X'Pert PRO X-ray diffractometer with Cu Kα radiation (*λ *= 0.154 nm) at a rate of 0.002° 2*θ *s^-1^, which was operated at 40 kV and 40 mA. TEM images and selected area electron diffraction (SAED) patterns were performed by a JEOL JEM-2010 (HT) transmission electron microscope operated at 200 kV, the samples were dissolved in ethanol and dropped directly onto the carbon-covered copper grids. SEM analysis of the samples was carried out with a FEI SIRION FESEM operated at an acceleration voltage of 25 kV. The BET surface area of the sample was measured by nitrogen adsorption in a Micromeritics ASAP 2020 nitrogen adsorption apparatus. The samples were degassed before the measurement. Magnetic hysteresis loops of samples were performed in Quantum Design PPMS (Physical Property Measurement System) equipped with a vibrating sample magnetometer (VSM) at room temperature with the external field up to 15 kOe. ATR-FTIR spectra were performed on a Thermo Fisher Nicolet iS10 FT-IR.

## Results and discussion

Forced hydrolysis method has been widely used for preparing α-Fe_2_O_3 _NPs since the first study by Matijevic et al. [4] and Cornell and Schwertmann [27]. In general, in the presence of water, the Fe^3+ ^salt dissociates to form the purple, hexa-aquo ion, the electropositive cations induce the H_2_O ligands to act as acids (except at very low PH) and hydrolysis by heating. In addition, the Fe salt was added to preheated water in order to avoid nucleation of geothite during the initial heating stage [4,28]. The synthesis of Fe_3_O_4 _NPs can be reached by reduction of α-Fe_2_O_3 _NPs in hydrogen atmosphere. In brief, the whole experimental process can be described as follows [4]:

(1) $${\text{FeCl}}_{\text{3}}\;+\;{\text{6H}}_{\text{2}}\text{O}\;\to \;\text{Fe}{\left({\text{H}}_{\text{2}}\text{O}\right)}_{\text{6}}{}^{\text{3}+}+\;{\text{3Cl}}^{-}$$

(2) $$\text{2Fe}{\left({\text{H}}_{\text{2}}\text{O}\right)}_{\text{6}}{}^{\text{3}+}\;\to \;{\text{Fe}}_{\text{2}}{\text{O}}_{\text{3}}\;+\;{\text{6H}}^{+}\;+\;{\text{9H}}_{\text{2}}\text{O}$$

(3) $${\text{3Fe}}_{\text{2}}{\text{O}}_{\text{3}}\;+\;{\text{H}}_{\text{2}}\;\to \;{\text{2Fe}}_{\text{3}}{\text{O}}_{\text{4}}\;+\;{\text{H}}_{\text{2}}\text{O}$$

In the hydrolysis process, the features that affect the products of the experiment generally include additive, reaction temperature, aging time, PH value. On the basis of previous reports, the addition anions have great effect on the shape of α-Fe_2_O_3 _NPs. The used PO_4_^3- ^anions will adsorb onto the crystal planes parallel to the *c*-axis of α-Fe_2_O_3_, which causes the growing of the α-Fe_2_O_3 _NPs along the c-axis direction and promotes the formation of spindle-like α-Fe_2_O_3 _NPs [22,29,30]. More detailed formation mechanisms in this study are currently under way.

**Figure 1:**
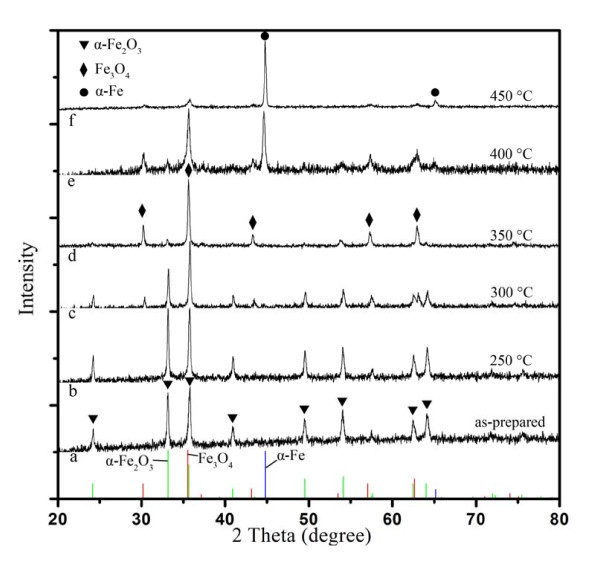
**XRD patterns of the samples S1 (a), S2 (b), S3 (c), S4 (d), S5 (e), and S6 (f)**.

Figure 1 shows the XRD patterns of the samples. Curve a is the pattern of S1. The diffraction peaks (2*θ *= 24.1°, 33.2°, 35.6°, 40.9°, 49.5°, 54.1°, 62.4°, and 64.1°) are coincided well with the value of JCPDS card 33-0664 (shown as green lines in the bottom), which could be well indexed to the pure hexagonal phase of hematite ((012), (104), (110), (113), (024), (116), (214), and (300)). Curve b displays the diffraction peaks of S2 (250°C). In this curve all the peak positions do not change, which reveals that the sample is still in α-Fe_2_O_3 _phase after annealing at this temperature. However, when the annealing temperature elevates to 300°C (S3), some new peaks (2*θ *= 30.2°, 43.3°, 57.3°, and 62.8°) are appeared in curve c. These peaks can be indexed to cubic spinel magnetite (JCPDS card 19-0629, indexed with red lines in the bottom). Moreover, the peaks of α-Fe_2_O_3 _become weak, which implies that the α-Fe_2_O_3 _NPs partially transform to Fe_3_O_4 _NPs after annealing at 300°C. Subsequently, all the peaks in the pattern of S4 (350°C) could be attributed to Fe_3_O_4_, their intensity become much stronger. The peaks attribute to α-Fe_2_O_3 _are almost disappeared, which demonstrates that the NPs is mainly Fe_3_O_4 _NPs. When the temperature was increased to 400°C (S5, shown in curve e), the peaks (2*θ *= 44.7°, and 65.0°) can be attributed to α-Fe (JCPDS card 06-0696, shown as blue lines in the bottom). Finally, the sample of S6 mainly transforms to α-Fe phase after annealing at 450°C (curve f).

**Figure 2:**
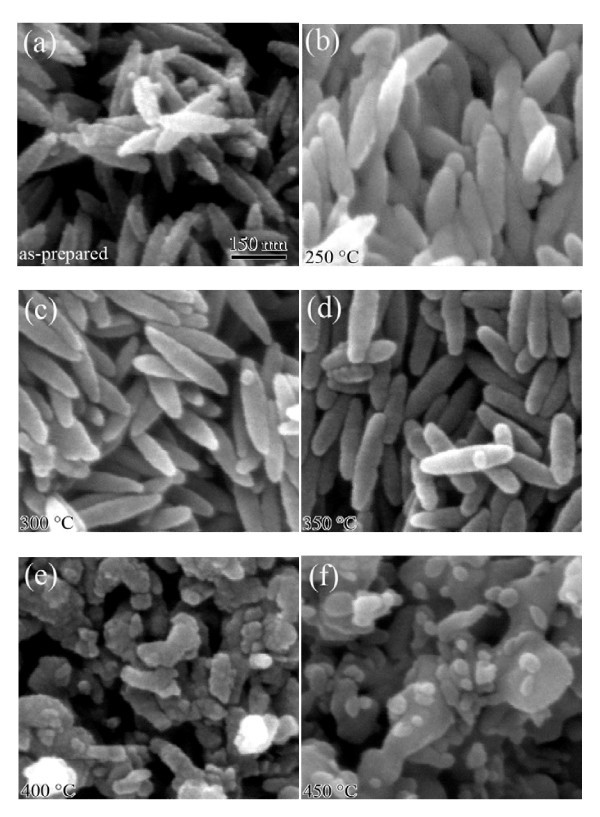
**SEM images of the samples S1 (a), S2 (b), S3 (c), S4 (d), S5 (e), and S6 (f)**.

The morphologies of the samples were studied by SEM analysis. The SEM image of S1 in Figure 2a clearly shows the formation of uniform spindle-like α-Fe_2_O_3 _NPs with the length and outer diameter approximately 250 and 60 nm, respectively. It is obvious that each of the spindle-like particles possesses a rough surface composed of many small particles. Figure 2b,c,d,e,f shows the SEM images of S2, S3, S4, S5, and S6, respectively. In the Figure 2b,c,d, their particle shape and size are preserved well. However, as shown in Figure 2e, when the annealing temperature increases to 400°C, the shape of the particles is damaged and many particles are melted. For the sample annealed at 450°C (shown in Figure 2f), the spindle-shape of precursor α-Fe_2_O_3 _NPs is disappeared completely. Instead, the obtained particles have irregular morphology. All the XRD and SEM results clearly indicate that α-Fe_2_O_3 _NPs can be transformed to Fe_3_O_4 _NPs after annealing in the reducing atmosphere with temperature up to 350°C, meanwhile the shape and size of the NPs are kept.

**Figure 3:**
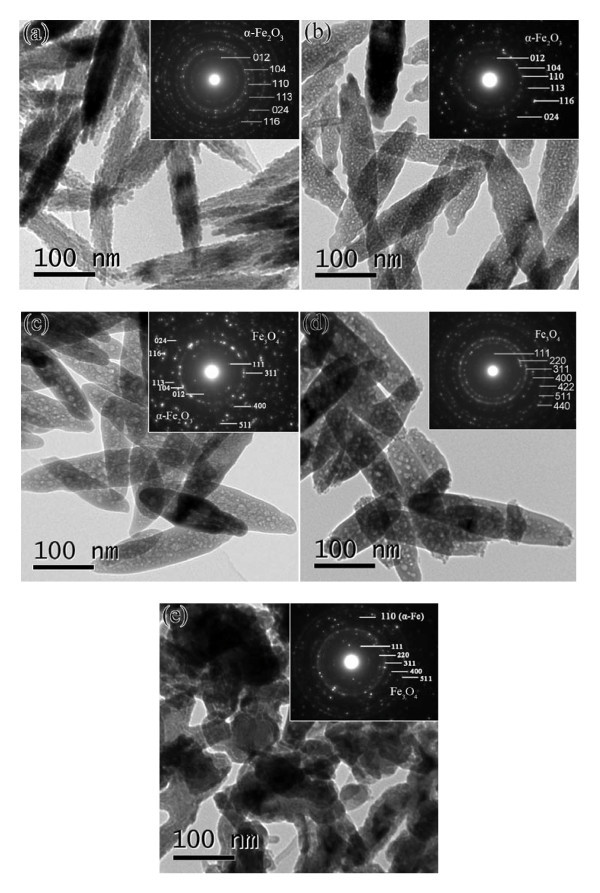
**TEM images and corresponding SAED patterns of samples S1 (a), S2 (b), S3 (c), S4 (d), and S5 (e)**.

For further discussing the morphologies and structures of the samples, TEM images of S1, S2, S4, and S5 are presented, as shown in Figure 3. It can be found in Figure 3a that the as-prepared α-Fe_2_O_3 _NPs are consisted of smaller closely packed particles, which causes rough surfaces. The inserted SAED pattern is in agreement with the structure plane of α-Fe_2_O_3_, which also reveals that the α-Fe_2_O_3 _NPs are in polycrystal. The TEM image of S2 in Figure 2b clearly illustrates that the NPs are mesoporous structure. The SAED pattern demonstrates that the sample is also in polycrystal feature with α-Fe_2_O_3 _phase. The results reveal that the porous structure has been formed after annealing at 250°C. Figure 3c shows the TEM image of S3 annealed at 300°C. It can be clearly seen that the shape and size of the particles are well preserved. Moreover, the size of the pores in the sample becomes larger than that of the pores in S2. This is because more vacancies are produced after reducing by H_2_. These vacancies aggregate to form larger pores. The inserted SAED pattern implied that the sample S3 is a compound of Fe_3_O_4 _and α-Fe_2_O_3_, which coincides with the XRD result. Figure 3d displays the TEM images of S4 (350°C). Although the sample S3 and S4 have similar porous structure, the SAED patterns of the samples are changed and the ring patterns of S4 can be indexed as a cubic spinel phase of magnetite, which demonstrates that the sample S4 are in Fe_3_O_4 _phase. Figure 3e shows the TEM images of S5. Clearly, some particles are also spindle-like and porous in structure. However, most of the particles are irregularly shaped, meaning that the shape of the sample has been partly damaged after annealing temperature at 400°C. This may be due to the collapse of NP structure, which is because too many large pores are produced inside the NP. The inserted SAED patterns reveal that the sample is a compound of Fe_3_O_4 _and α-Fe. The TEM result is in good agreement with the XRD and SEM results. Moreover, it proves that the annealing treatment can cause the mesoporous structure.

**Figure 4:**
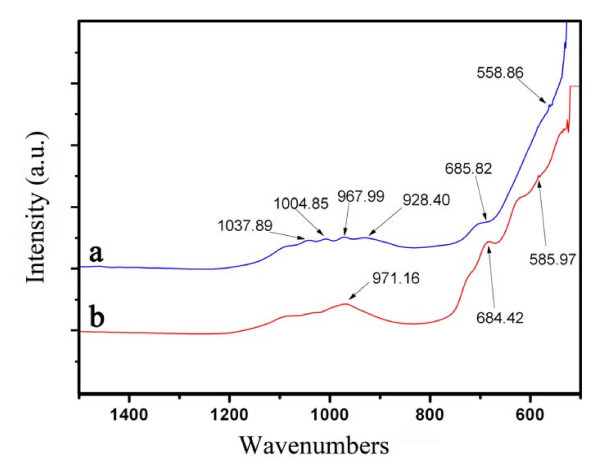
**ATR-FTIR spectra of α-Fe**_**2**_**O**_**3 **_**NPs (a) and Fe**_**3**_**O**_**4 **_**NPs (b)**.

Figure 4 shows the ATR-FTIR spectra of the samples S1 (a) and S4 (b). The absorption band at 558.86 cm^-1 ^in the curve a is attributed to the bending vibrations of the Fe-O in α-Fe_2_O_3 _[31], while the fingerprint bands at 1037.89, 1004.85, 967.99, and 928.40 cm^-1 ^could be related to PO_4_^3- ^anions [32]. In the curve b, there is an absorption band at 971.16 cm^-1^. This band is attributed to NaFePO_4 _[33], which indicates that a new component (NaFePO_4_) might be generated on the surface of the particles after annealing. The absorbtion band at 585.97 cm^-1 ^is associated with the Fe-O stretching mode of the Fe_3_O_4 _NPs [34-36]. In addition, the absorption band at about 685 cm^-1 ^is observed in both of the curves, which is assigned to the bending modes of Fe-O-H [31]. The ATR-FTIR results further prove the phase transformation of NPs from α-Fe_2_O_3 _to Fe_3_O_4_. Moreover, the detection of the phosphate reveals that the phosphate possibly plays an important role in the formation of the spindle and porous structures.

**Figure 5:**
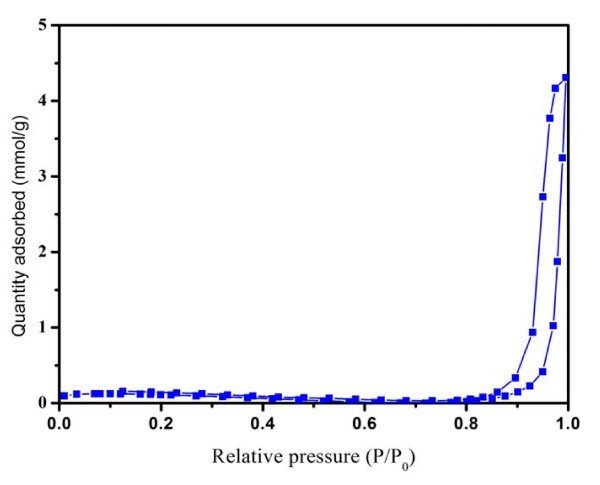
**N**_**2 **_**adsorption and desorption isotherms of Fe**_**3**_**O**_**4 **_**NPs**.

Nitrogen adsorption-desorption isotherms were performed to determine the surface area and pore size of S4, which is shown in Figure 5. The BET surface area is measured using multipoint BET method within the relative pressure (*P*/*P*_0_) range from 0.05 to 0.3. The pore size distribution was determined by the Barret-Joyner-Halender (BJH) method using desorption isotherm. The pore volume and average pore size for the sample were determined according to the nitrogen adsorption volume at the relative pressure (*P*/*P*_0_) of 0.9956. As shown, the sample exhibits a type H3 hysteresis loop according to Brunauer-Deming-Deming-Teller (BDDT) classification, which indicated the presence of mesopores (2-50 nm) with a cylindrical pore mode [37]. According to the BET method, the specific surface area of the samples is determined to be 7.876 m^2 ^g^-1^. The BJH adsorption cumulative volume of pores between 17 and 300 nm is 0.15 cm^3 ^g^-1^. However, the BJH adsorption average pore of the sample is 78.1 nm, which is probably because the pores in the particles are hermetic, nitrogen could not be contact with the internal wall of the pores [37]. On the other hand, the aggregation of the Fe_3_O_4 _NPs will cause many spaces among them, which can also lead to the larger result of the pore size [38,39]. The density of the sample based on the current BET result is calculated to be 2.16 g cm^-3 ^(Assuming that each Fe_3_O_4 _NPs is an ellipsoid, thus $\rho =\frac{M}{V}$, and *M *= *A*_s _· *S*, where *ρ *is the density of the sample; *M*, *S *and *V *are the mass, surface area and volume of one Fe_3_O_4 _particle, respectively; *A*_s _is the BET surface area of the sample. As $V=\frac{4}{3}\pi r_{a}r_{b}^{2}$ and $S=2\pi r_{b}\left(\frac{7}{3}r_{a}^{2}+\frac{2}{3}r_{a}r_{b}+r_{b}^{2}\right)$, where *r*_*a *_and *r*_*b *_are the length and outer diameter of the Fe_3_O_4 _NPs, the density of the sample based on the BET result is estimated to be 2.16 g cm^-3^), it is smaller than 5.18 g cm^-3 ^for corresponding bulk Fe_3_O_4_, which indirectly proves that the Fe_3_O_4 _NPs are in porous.

**Figure 6:**
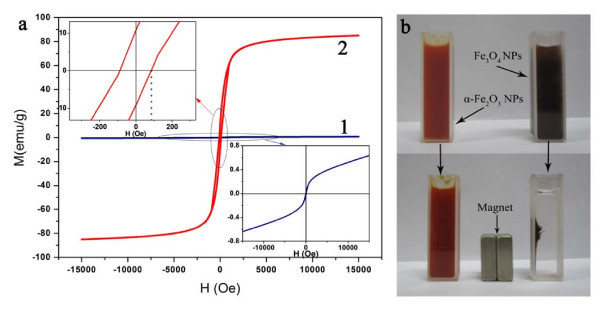
**Magnetic hysteresis loops of α-Fe**_**2**_**O**_**3 **_**NPs (curve 1) and Fe**_**3**_**O**_**4 **_**NPs (curve 2) (a); photographs of α-Fe**_**2**_**O**_**3 **_**NPs and Fe**_**3**_**O**_**4 **_**NPs before and after magnetic separation with an external magnetic field (b)**.

As the physicochemical properties of samples are related to their morphologies and structures, the magnetic hysteresis loops of the samples (S1 and S4) were measured by VSM at room temperature, and the results are shown in Figure 6a. From the curve 1, we can see that the sample exhibits weak ferromagnetic behavior before annealing, and its saturation magnetization and coercivity are 0.64 emu g^-1 ^and 37.6 Oe, respectively. It has been proved that the structure of α-Fe_2_O_3 _can be described as consisting *hcp *arrays of oxygen ions stacked along the [001] direction. Two-thirds of the sites are filled with Fe^3+ ^ions, which are arranged regularly with two filled sites being followed by one vacant site in the (001) plane thereby forming sixfold rings. In this case, the arrangement of cations produces pairs of Fe(O)_6 _octahedra, and Fe^3+ ^ions are antiferromagnetically coupled across the shared octahedral faces along the c-axis. In the basal plane, there are two interpenetrating antiferromagnetic sublattices. As the electron spins of these sublattices are not exactly antiparallel (with a canting angle of <0.1°), a weak ferromagnetic interaction is resulted, and this effect dominates the magnetic behavior at room temperature [4]. As shown in curve 2 (Figure 6a), the S4 possessed a saturation magnetization of 85.18 emu g^-1 ^and a coercivity of 86.01 Oe, the saturation magnetization is close to 92 emu g^-1 ^for corresponding bulk Fe_3_O_4 _[40], which is because the α-Fe_2_O_3 _phase of the NPs has transformed to Fe_3_O_4 _phase after annealing. The structure of magnetite is inverse spinel, where there is a face-centered cubic unit cell based on 32 O^2- ^ions which are regularly cubic close packed along the [111]. Two different cation sites occupied by Fe^2+ ^and Fe^3+ ^form two interpenetrating magnetic sublattices. At room temperature the spins on the two sites are antiparallel and the magnitudes of types of spins are unequal, which causes the ferromagnetism of magnetite. In addition, the particle size and crystal morphology affect the coercivity in the order: spheres < cubes < octahedral in line with the increase in the number of magnetic axes along this series of shapes [4]. In addition, anisotropy shape of the particles may also affect the magnetism [41]. Figure 6b shows the photographs of the samples dispersing in ethanol with and without an external magnetic field. It can be clearly seen that the Fe_3_O_4 _NPs are well dispersed in ethanol before magnetic separation. However, after magnetic separation all Fe_3_O_4 _NPs are attracted together by magnet. And the separating time only needs 35 s. For comparison, the α-Fe_2_O_3 _NPs dispersing in ethanol almost do not change before and after magnetic separation. The results demonstrate that the Fe_3_O_4 _NPs present excellent magnetic separation property and have good potential application for recyclable nanomaterials.

## Summary

In conclusion, spindle-like α-Fe_2_O_3 _NPs were fabricated by forced hydrolysis of FeCl_3 _in the presence of PO_4_^3- ^anions. The as-prepared α-Fe_2_O_3 _NPs were then reduced in hydrogen at 350°C and transformed into spindle-like Fe_3_O_4 _NPs with mesoporous structure. The as-obtained mesoporous Fe_3_O_4 _NPs possess a high BET surface area of 7.876 m^2 ^g^-1^. In addition, the obtained Fe_3_O_4 _NPs possessed a high saturation magnetization of 85.18 emu g^-1 ^and a coercivity of 86.01 Oe. Owing to its excellent magnetic separation property and special mesoporous structure, the as-obtained Fe_3_O_4 _NPs may have a great potential application in the future.

## Abbreviations

AP: analytically pure; ATR-FTIR: attenuated total reflection fourier transform infrared spectroscopy; BDDT: Brunauer-Deming-Deming-Teller; BET: Brunauer-Emmett-Teller; BJP: Barret-Joyner-Halender; FSEM: field emission scanning electron microscopy; MRI: magnetic resonance imaging; NPs: nanoparticles; SAED: selected area electron diffraction; TEM: transmission electron microscopy; VSM: vibrating sample magnetometer; XRD: X-ray diffraction.

## Competing interests

The authors declare that they have no competing interests.

## Authors' contributions

SZ participated in the materials preparation, data analysis and drafted the manuscript. WW, XX and JZ participated in the sample characterization. FR conceived and co-wrote the paper. CZ participated in its design and coordination. All authors read and approved the final manuscript.
